# Colour Stabilization of Oak, Spruce, Larch and Douglas Fir Heartwood Treated with Mixtures of Nanoparticle Dispersions and UV-Stabilizers after Exposure to UV and VIS-Radiation

**DOI:** 10.3390/ma11091653

**Published:** 2018-09-07

**Authors:** Miloš Pánek, Eliška Oberhofnerová, Štěpán Hýsek, Přemysl Šedivka, Aleš Zeidler

**Affiliations:** Department of Wood Processing and Biomaterials, Faculty of Forestry and Wood Sciences, Czech University of Life Sciences Prague, Kamýcká 129, 165 00 Prague, Czech Republic; oberhofnerova@fld.czu.cz (E.O.); hyseks@fld.czu.cz (Š.H.); sedivka@fld.czu.cz (P.Š.); zeidler@fld.czu.cz (A.Z.)

**Keywords:** wood, colour stabilization, accelerated ageing, surface treatment

## Abstract

Colour changes and associated wood degradation in exterior and interior applications influenced by ultraviolet (UV) and visible radiation (VIS) decreases the aesthetic value of the products and shortens the overall life of transparent coatings. The aim of the paper is to achieve colour stabilization of oak, larch, Douglas fir and spruce heartwood via surface treatment with UV stabilizers, hindered amine light stabilizers (HALS), nanoparticles TiO_2_, ZnO, and mixtures thereof, during exposure to UV and VIS radiation. Colour changes were evaluated during accelerated artificial ageing testing in Xenotest. The distinctly individual character of colour changes in surface treatments due to the underlying wood species was confirmed. A synergistic effect was found when using a combination of active substances compared to substances used individually. The mixture of benzotriazoles with HALS (Tinuvin 5151) in combination with TiO_2_ and ZnO nanoparticles was confirmed as one of the most effective treatments for colour stabilization of wood due to UV and VIS spectrums.

## 1. Introduction

Preservation of the original appearance of wood exposed to outdoor conditions is still an unresolved problem [1,2], and reducing and slowing colour changes is still unsatisfactory [3,4], also in interior applications [5]. It is not just a question of design or structural solutions [6,7], but the reason why naturally coloured wood has a positive influence on well-being [8].

Wood used outdoors (use class 3 in EN 335 [9]) is exposed to more degrading effects compared to indoors (use class 1 in EN 335 [9]). In the first stage the decomposition of lignins and extractives is carried out due to photodegradation reactions and an initial darkening of wood is observed. Lignin photodegradation is connected with the creation of phenoxy radicals, which are transformed into quinoid structures. These structures cause the yellowing of the wood surfaces [10]. Subsequent leaching of photodegraded products by precipitation is connected with an observed lightening of the wood. The penetration of dirt particles [11], mould [12], and wood-staining fungi causes the greying of wood [13]. Interior photodegradation without liquid water action leads to the darkening and turning of wood surfaces to yellow and red shades [5] because photodegraded lignins and extractives are not leached by rainwater.

Currently, the most commonly used variant of the protection of native wood surface layers against weathering [14] is the application of transparent coatings [1]. Even here, however, it is necessary to expect a significant colour change in the underlying wood [10], film of coating [15,16] or UV-stabilizing microparticles [17] and a shorter overall lifetime of transparent compared to pigmented coatings [18].

Wood modifications for bio-resistance improvement [19], e.g. acetylation, thermal, have provided unsatisfactory results against weathering thus far, as modified wood exposed to the elements also turns grey [20]. The possibilities of hydrophobic and self-cleaning coatings are still limited due to the fact that their long-term durability on wood in harsh conditions is not ensured [21,22]. 

The colour stability of the wood under the transparent coating is also required for products in the interior (furniture, parquet, cladding, etc.) and has been researched in more works [16,23]. UV radiation causes pronounced colour changes in coated wood relatively fast in the interior [24]. Due to the uneven impact of sunlight over the window glass, different colour changes can occur in different parts of the room. Even this problem can be solved by the effective stabilization of underlying wood against UV and VIS spectra.

**Figure 1:**
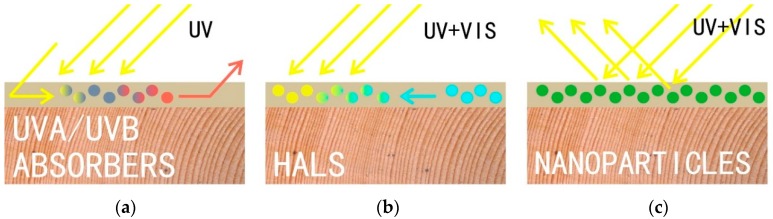
Schematic representation of the principle of the effect of UV-absorbers (**a**) HALS (**b**) and nanoparticles (**c**) for reducing the degradation effect of solar radiation on surface layers of wood. (**a**) Radiation absorbed and converted into heat energy by UV absorbers [25,28,33,47]; (**b**) Harmful free radicals absorbed and neutralized by HALS [26,42]; (**c**) Radiation reflected and scattered by nanoparticles [27,33,45,48].

Wood colour stabilization using surface treatment by UV-stabilizers [25], HALS [26], and nanoparticles [27] on which the top transparent coating [28] can be applied, is an explored variant. This path seems promising because it combines the slowing of lignin and the extract decomposition (Figure 1) associated with colour changes [29,30,31,32] and the protective function of the top coating. In addition, the top coating may contain UV-stabilizers, HALS, or nanoparticles [26,28,33,34,35,36,37,38]. Therefore, the overall efficacy against UV radiation and VIS spectra is multiplied. In addition, a top hydrophobic layer can be included in the layering, which reduces the synergistic effect of rainwater on the degradation of the coating system during long-term exposure [4,18]. 

A number of works have focused on surface treatment of surface layers of wood for decreasing the effect of UV and VIS-spectra [5,39]. UV-stabilizers, HALS [10,40,41], a combination thereof [1,42] and nanoparticles [27,43,44,45] were all utilized. The principles of their effectiveness are described in many works [10,26,27,33]. UV-stabilizers absorb UV light mainly in the spectral range between 300 nm and 400 nm. The most used are 2-(2-hydroxyphenyl)-benzotriazoles and 2-hydroxyphenyl-s-triazine [26]. HALS have the ability to trap free radicals, which are created during the decomposition of lignin into phenoxy radicals [10] under the action of visible spectra [46]. Mineral nanoparticles absorb and reflect UV and VIS spectra of radiation [33]. More effective is increasing the active area using smaller nanoparticles, which also allows us to improve the transparency of protective layers [26,27].

The results are variable, but the use of a combination of UV-stabilizers and HALS [10,42] and TiO_2_ or ZnO nanoparticles [49,50,51] seems to be the most effective. In addition, it is necessary to consider the fact that the kind of underlying wood species, and in particular the content of specific extractives [52], significantly affects the reaction to the impacting UV radiation [53,54,55] and also reacts differently with the additives and polymeric foundations contained in the coatings [4,33,56,57].

**Figure 2:**
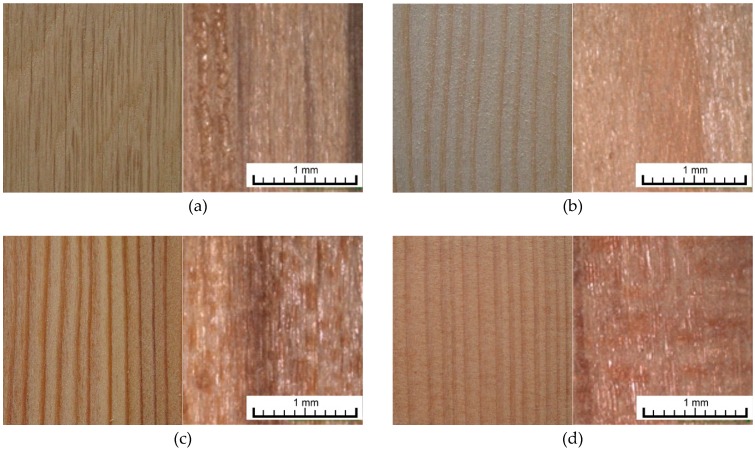
Photos of surfaces (left) and confocal laser scanning microscopy images (right) of wood species used in experiment (see also Table 1). (**a**) English oak; (**b**) Norway spruce; (**c**) European larch; (**d**) Douglas fir.

One representative of hardwoods (English oak) and three softwoods (European larch, Douglas fir and Norway spruce) were tested in this experiment (Figure 2). Oak wood is widely used for interior applications (furniture, parquets, windows and doors), but, due to its high durability (EN 350 [58]), it also appears in exterior wooden constructions (bridges, garden constructions and furniture, balconies, terraces, etc.). It contains a relatively high amount of phenol extractives, mainly vescalagin, castalagin, gallic and ellagic acids [54]. Surface treatment is problematic due to phenols’ role in coatings’ durability [59]. The photodegradation process and colour changes are fast, mainly in the heartwood zone [10], and leaching of extractives is observed [21].

Larch wood is popular for exterior applications (mainly for claddings, windows, decking or fences) due to the relatively high durability against fungi and insects (EN 350) and attractive appearance (Figure 2). Logs contain only a small amount of non-durable sapwood zone. The heartwood zone is rich in extractives, mainly arabinogalactanes, terpenes and terpenoids [60]. Problems with transparent coatings application have also been reported, though [61]. The Douglas fir is a domestic species widely used in North America but introduced to Europe due to its good adaptability to climate changes in recent decades [62]. The wood is relatively durable (EN 350 [58]) and rich in extractives—flavonoids, phenolic acid derivates and terpenes [63]. Spruce wood is most commonly used for exterior and interior construction and products due to its relatively low cost and good density/stiffness ratio. The most important disadvantage is its susceptibility to bio-attack (EN-350 [58]) and bad adaptability to climate change—mainly in Central Europe [64]. This will probably result in less availability in the next decades.

The main aim of this experiment is to determine the effectiveness of selected UV-stabilizing solutions and dispersions containing UV-stabilizers, HALS or nanoparticles of TiO_2_ and ZnO for reducing the colour changes in four types of tree species’ heartwood (oak, larch, spruce, Douglas fir) under the influence of accelerated artificial ageing in a Xenon chamber. One of the objectives and the novelty of this work was to find the potential synergic effects of mixtures of these substances; therefore, their various combinations were tested. Our other tested hypothesis was the influence of the kind of underlying wood on the effectiveness of colour-stabilizing surface treatments.

## 2. Material and Methods 

### 2.1. Wood Samples

Test samples from four types of tree species were used (Figure 2): one hardwood, English oak (*Quercus robur*, L.), and three softwoods, Norway spruce (*Picea abies*, L. Karst), European larch (*Larix decidua*, Mill.) and Douglas fir (*Pseudotsuga menziesi*, (Mirb), Franco) from trees cut down in forest stands of the Forest Establishment of the Rychnov nad Kněžnou region, approximately 130 km east of Prague, in the Czech Republic. The samples were collected in Prague, in the Czech Republic. The area of growth is characterised by an average annual temperature of 7.9 °C, an average total annual precipitation of 561.2 mm, and an average growing season of 140–150 days. Only samples from heartwood were tested. Samples were prepared from different trees (the age of trees ranged from 100 to 120 years) and planks. The heartwood zone was selected visually. The visual selection of tested samples was done in line with our previous work [21] to avoid differences in the initial colour of wooden surfaces. The average data on the tested tree species and test samples are specified in Table 1.

The dimensions of the test samples were 45 mm × 45 mm × 20 mm (45 mm in longitudinal direction), and four specimens were used for each of the used surface treatments.

### 2.2. Surface Treatments

In addition to two stabilization treatments, i.e., by Sun Care 800 and 900 (T10 and T11—see Table 2), 3 wt % of the total concentration of active components in solutions, dispersions or combinations thereof (Table 2) were used for surface treatments. There were more reasons to use 3% concentrations of active compounds. Lower concentrations are not sufficient to protect wooden surfaces against UV and VIS radiation [27,40,42]. On the other hand, our preliminary laboratory tests showed that higher concentrations of TiO_2_ and ZnO increase the colour changes of wood rich in extractives after their application. In order to achieve a uniform and compact penetration layer, the surface materials were applied by spraying on a clean wood surface with 8% relative humidity at (22 ± 0.5) °C, treated with sandpaper with a grain size of 120. The surface treatment was always applied in one layer on the radial surfaces of larch, Douglas fir, spruce (narrower latewood zones in comparison with tangential surfaces) and tangential surfaces of oak wood (narrower parenchyma rays in comparison with radial surfaces) samples (Figure 1). This allows us to decrease the effect of non-homogenous anatomical structure of tested woods during colour measurements using spectrophotometer with d/8 geometry (Section 2.5).

Only the stabilization of the underlying wood against UV and VIS spectra was solved by using the penetration layer in this work and no topcoat was applied. The reason is that for various uses, indoor and outdoor, it is necessary to use different top coating systems with specific resulting properties. Appropriate stabilization of the underlying wood colour would provide a universal solution for the application of more types of top coating systems.

### 2.3. Accelerated Ageing

Artificial ageing was simulated in Xenotest Q-Sun Xe-3 (Q-Lab, Cleveland, OH, USA) using methods based on the work of Kataoka and Kiguchi [66] and by applying a Daylight glass filter-Q (Daylight-Q). The testing parameters were: temperature of air in the chamber, 45 °C; black panel temperature, 60 °C; water spraying was off; UV irradiance between 300 nm and 400 nm; (TUV) 41 W·m^−2^; relative air humidity of 30%. The UV glass filter simulated outdoor conditions, with a higher impact of UV and VIS radiation in comparison with indoor tests using window glass filter [5,46,67]. Water spraying was not used because the tested colour stabilization of wooden surfaces only decreases the degradation of wooden surfaces and improves the durability of the transparent topcoat, as was mentioned in the work of Evans et al. [1]. The use of a topcoat is necessary to reduce the leaching of active UV-stabilizing substances on the wooden surface [21].

Colour changes in test specimens were assessed after 50-, 160- and 320-h experiments. The total amount of energy (UV, IR, VIS-spectra) that hit the test samples in the Xenotest during the course of 320 h of artificial accelerated ageing was 47,864 kJ·m^−2^.

### 2.4. Microscopic and Elemental Composition Analyses

Both the surface and the section of the penetration layers on the surfaces of treated samples were observed with a MIRA 3 electron microscope (Tescan Orsay Holding, Brno, Czech Republic) with a secondary electron detector operated at 15 kV acceleration voltage. The samples for scanning election microscopy were cut to obtain transverse and tangential surfaces using a GSL1-microtome; the section thickness was 50 µm. Specimens were then gold coated by the coater Q150R ES (Quorum Technologies Ltd., East Sussex, UK). The used working distance was 15 mm and the spot size was 5 nm. The vacuum mode was set as follows: gun pressure, 1.2 × 10^−8^ Pa; column pressure, 6.9 × 10^−4^ Pa; chamber pressure, 8.3 × 10^−2^ Pa. The elemental composition of the surface and the section of the penetration layer were examined by an energy-dispersive spectroscopy system (Bruker XFlash X-ray detector, Karlsruhe, Germany, and ESPRIT 2 software, Camarillo, CA, USA). Hydrogen is not detectable by the method used.

Microscopic structural surface characteristics of tested wood species were additionally observed under 108-fold magnification with a confocal laser scanning microscope Lext Ols 4100 (Olympus, Tokyo, Japan).

### 2.5. Colour Analyses

The colour of the tested samples was evaluated in the native state, after application of surface treatments and during the accelerated ageing in Xenotest after 50, 160 and 320 h. The equipment used was Spectrophotometer CM-600d (Konica Minolta, Osaka, Japan). Set observation angle was 10°, d/8 geometry, D65 light source and the SCI method were used. Sixteen measurements per type of tested samples were carried out at the same place in the sample. The evaluation of colour change was done in a CIE-L*a*b* colour system and changes in L*, a*, and b* colour components were evaluated: L* = lightness (0-black to 100-white), a* = chromaticity coordinate (+ red or − green), and b* is chromaticity coordinate (+ yellow or − blue). The colour coordinates changes (∆L*, ∆a*, and ∆b*) between the treated or aged samples and their initial state were determined. The total colour difference ∆E* [52] was evaluated using Equation (1):

(1) $$\;\Delta E^{*}=\sqrt{{\left(\Delta L^{*}\;\right)}^{2}+{\left(\Delta a^{*}\right)}^{2}+{\left(\Delta b^{*}\right)}^{2}},$$

where:

∆E_AP_*—The total colour difference after surface treatment application
∆E_50_*—Change in the original colour after 50 h of artificial ageing (due to the original colour of the wood without surface treatment)
∆E_160_*—Change in the original colour after 160 h of artificial ageing (due to the original colour of the wood without surface treatment)
∆E_320_*—Change in the original colour after 320 h of artificial ageing (due to the original colour of the wood without surface treatment)

### 2.6. Roughness Measurements

The surface roughness of the tested wooden samples was determined using a profilometer Talysurf Form Intra (Taylor-Hobson, Leicester, UK) in accordance with EN ISO 4287 [68] and EN ISO 4288 [69]. In total 16 measurements per type of evaluated set was performed over the samples’ surface, perpendicularly to their length. Average mean roughness (*Ra* in µm), which represents the arithmetical mean of the absolute ordinate values within a sampling length, was measured*.*

### 2.7. Statistical Analyses

Data were analysed using MS Excel and STATISTICA 13.2 (StatSoft, Palo Alto, CA, USA). Mean values, standard deviations, HSD Tukey-test and multifactorial ANOVA was used for analysing the statistical significance of selected factors using the 95% significance level. Mean values of total colour changes after application of surface treatment and after 50, 160 and 320 h of accelerated ageing were used in histograms. Surface roughness changes were displayed by whisker plots. 

## 3. Results and Discussion

There exist a number of methods for decreasing the colour changes in underlying wood species by modification of surface layer treatments, as summarised in the works of George et al. [10], Schaller and Rogez [42] and Evans et al. [1]. This experiment was focused on the penetration treatment of underlying wood species with UV stabilizers, HALS, nanoparticles, or combinations thereof. For comparison, two commercial products were tested for the penetration of underlying wood species (T11, T12—see Table 2)—the results of total colour changes are shown in the Section 3.1.

**Figure 3:**
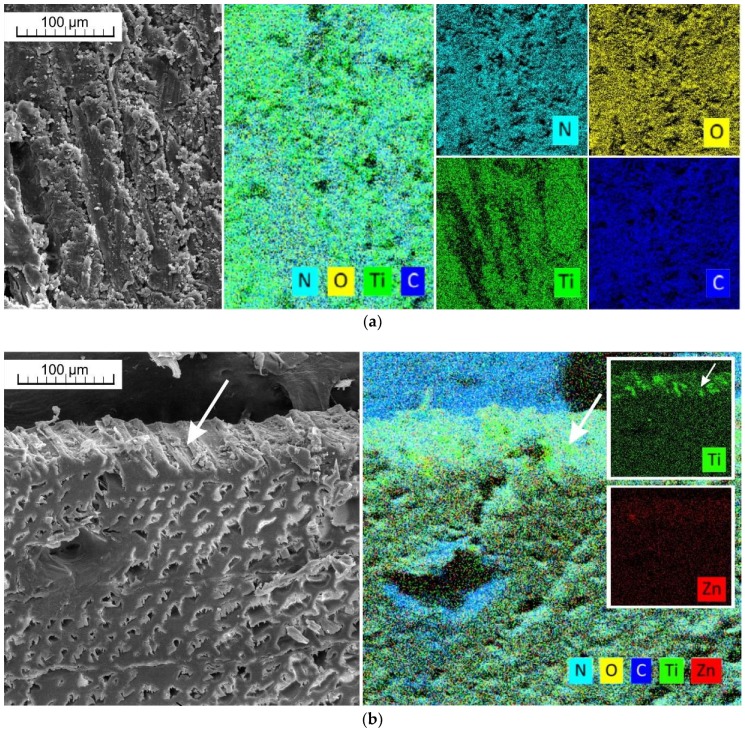

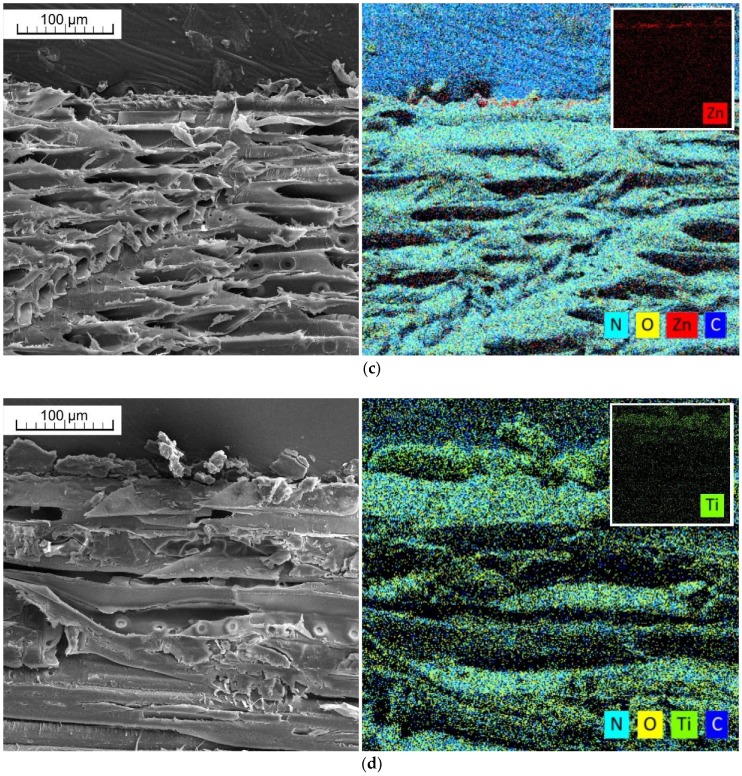
SEM and elemental microscopic analyses of surface treated wood. (**a**) Surface of larch wood treated by T7 formulation—nitrogen (N) is a compound of HALS and titanium nanoparticles (Ti) was applied as TiO_2_; (**b**) Cross section of larch wood samples—a combination of zinc nanoparticles (Zn) and titanium nanoparticles (Ti) was applied as treatment T5, deposited only on the surface of the larch wood tracheids; (**c**) Zinc nanoparticles (Zn) applied as ZnO using treatment T6 are deposited only on the surface of the larch wood tracheids; (**d**) Titanium nanoparticles (Ti) applied in treatment T7 are deposited only on the surface of the larch wood tracheids.

Microscopic and elemental composition analyses have shown that UV and VIS protective treatment penetrate only to the surface of cell elements destroyed during sanding (see white arrows in Figure 3b); deeper penetration into wood was not achieved (Figure 3a–d). This was mainly caused by the very poor permeability of the tested wood species [58] and the non-pressure technique of surface impregnation.

### 3.1. Colour Changes

**Figure 4:**
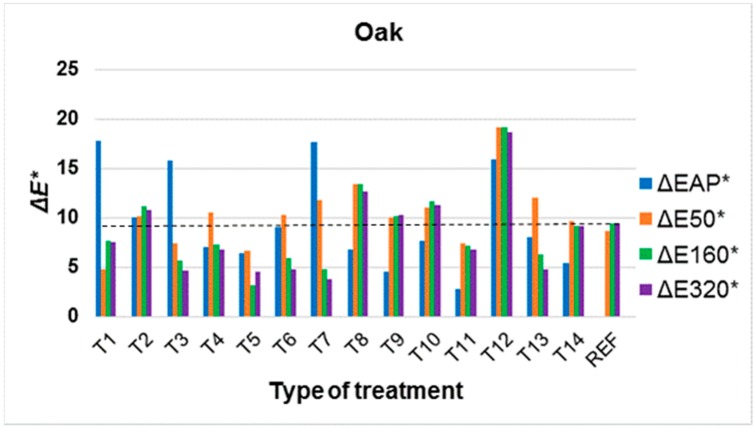
Colour changes in oak wood after application of surface treatment (ΔEAP*) and after 50, 160 and 320 h (ΔE50*, ΔE160*, ΔE320*) of accelerated ageing. (All evaluated total colour changes ΔE* in graph were compared to untreated wood surface; the dotted line in the graphs shows the comparison of ΔE* after 320 h of ageing between untreated samples (REF) and treated samples (T1–T14).)

**Figure 5:**
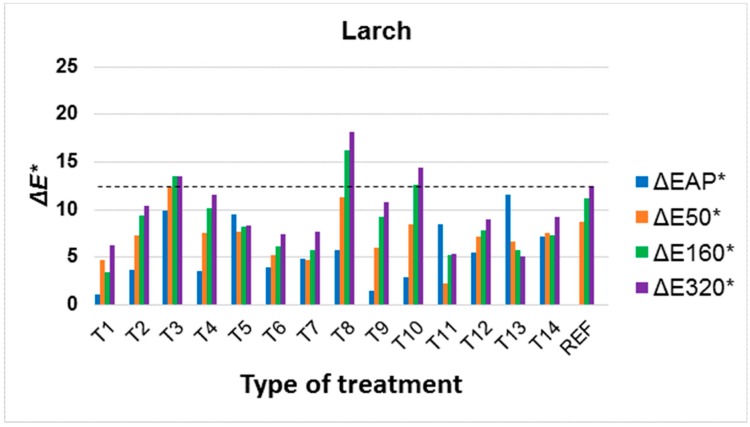
Colour changes in larch wood after application of surface treatment (ΔEAP*) and after 50, 160 and 320 h (ΔE50*, ΔE160*, ΔE320*) of accelerated ageing. (All evaluated total colour changes ΔE* in graph were compared to untreated wood surface.)

**Figure 6:**
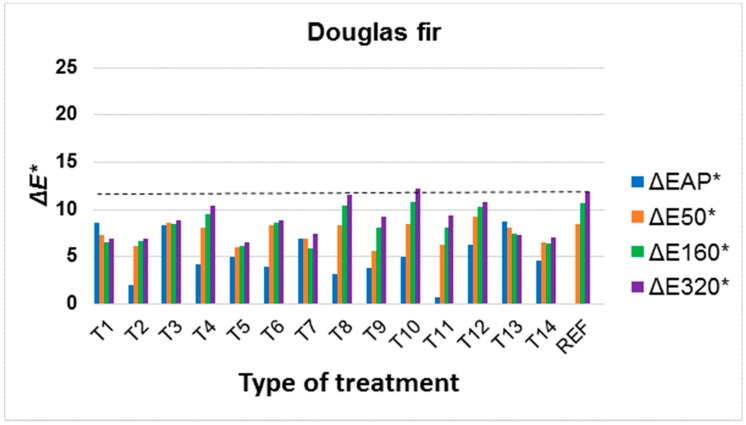
Colour changes in Douglas fir wood after application of surface treatment (ΔEAP*) and after 50, 160 and 320 h (ΔE50*, ΔE160*, ΔE320*) of accelerated ageing. (All evaluated total colour changes ΔE* in graph were compared to untreated wood surface.)

**Figure 7:**
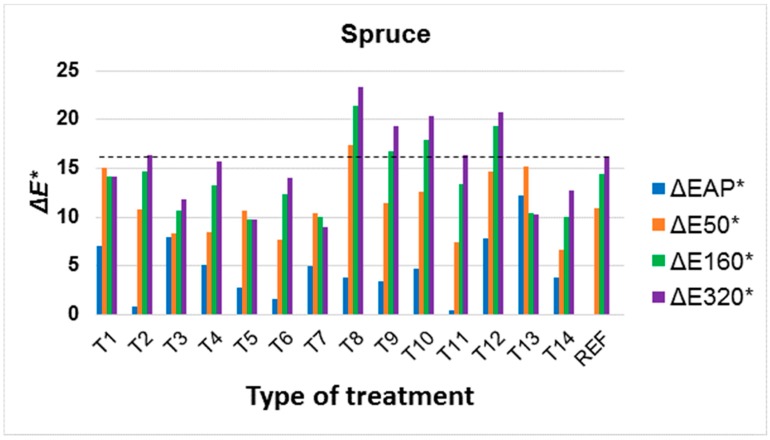
Colour changes in spruce wood after application of surface treatment (ΔEAP*) and after 50, 160 and 320 h (ΔE50*, ΔE160*, ΔE320*) of accelerated ageing. (All evaluated total colour changes ΔE* in graph were compared to untreated wood surface.)

Colour changes in modified surfaces were evaluated during artificial ageing in relation to the original colour of the underlying wood species, as it is a supportive idea to preserve the original colours of the underlying wood species. However, the change to colour in the underlying wood species after application of the surface treatment (ΔEAP*) was also evaluated. It is clear from the results that the change in colour after application of the treatment, but also during ageing due to UV and VIS spectra, strongly depends on the underlying wood species (Figure 4, Figure 5 and Figure 6, Table 3 and Table 4). Better colour stabilization results are more difficult to achieve in oak (Figure 4) and spruce (Figure 7) compared to Douglas fir (Figure 6) and larch (Figure 5). With regard to larch (Figure 5), a more pronounced colour stabilization compared to the natural surface (REF) was achieved in most cases. 

**Figure 8:**
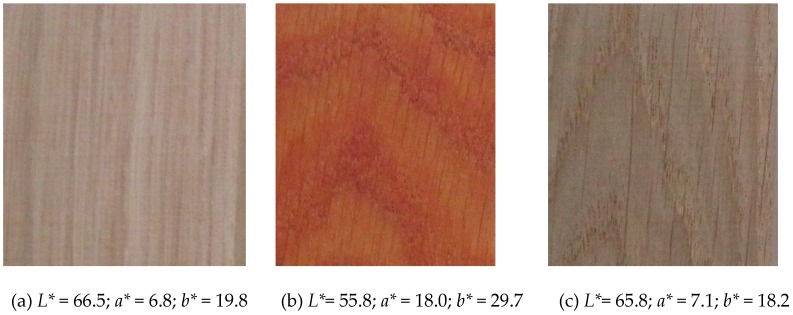
Photos of colour changes in oak wood (**a**) after application of surface treatment T1 with 3% concentration of nanoparticles TiO_2_ (**b**); and additional colour change after 4 h of accelerated ageing (**c**).

With regard to oak, successful treatment was hampered by a significant change in colour even after modification treatments were applied (Figure 4). This was most evident after the application of nanoparticles TiO_2_ (T1, T3). With regard to T1 on oak, an interesting phenomenon was observed wherein the reaction of extractives to nanoparticles TiO_2_ led to a significant change in colour to orange shades (Figure 8a,b). However, the impact of UV+VIS spectra, which lasted 4 h according to the configured parameters (see Section 2.3) and the impact of 590 kJ·m^−2^ energy on the surfaces of the specimens led to the partial degradation of extractives [54], which is very fast in the initial stages of photodegradation [55], and the return of the colour of treated specimens to approximately the original shades (Figure 8c). For several oak wood treatments (T3, T4, T6, T7, T13-Figure 4) a phenomenon was observed whereby artificial ageing reduced the overall colour changes. The continuous decomposition of extractive substances [54] likely led to a decrease in the reaction with surface treatment substances, although not as fast as with the T1 (Figure 8). For oak, efficient treatments that appear to stabilize colour are T5, T7 and T11 (Figure 4, Table 3). However, for treatment T7, there appears to be an unsuitable high colour change after the application of a mixture containing TiO_2_, the same as for the T1 (Figure 8). This effect was less significant for the T5 via a decrease on the proportion of TiO_2_ in the treatment layer, and variant T11 appears to be an advantage, wherein there was no significant colour change even after the application or during ageing (Figure 4, Table 3).

For the other tree species, after application, the colour change was not observed to such an extent (Figure 5, Figure 6 and Figure 7), but with regard to preserving the original appearance of the underlying wood species, this phenomenon must generally be expected for all types of tree species tested. For spruce, the difficulty of colour stabilization is primarily caused by the original light colour (Figure 2, Table 1), and thus by the greater change to darker and yellow, or red shades due to photodegradation [31]. Despite the use of a surface treatment, there was only a statistically significant decrease in colour changes in four (T3, T5, T7, T13) treatments after 320 h of artificial accelerated ageing (Table 3) compared to an untreated surface (Figure 7). However, the T13 treatment is limited by the high colour change in the surface after the application of the treatment. Treatments T3, T5 and T7 consisted of a treatment containing TiO_2_, which confirms the positive effect confirmed in a number of works [22,43,55]. The most effective colour stabilization in spruce was achieved via a combination of T7, where TiO_2_ was combined in a 1:1 ratio with Tinuvin 5151 (combination of HALS and benzotriazoles). 

In larch, T1, T6, T7 appear to be effective treatments, decreasing the colour change via UV and VIS spectra, where there are no significant colour changes after application or a decrease thereto during ageing. Once again, the stabilizing effect of TiO_2_ either alone (T1) or in combination with UV stabilizer and HALS (T7) was manifested, which is in line with works that directly tested the effect of TiO_2_ nanoparticles on other tree species [70,71]. A treatment containing a mixture of nanoparticles ZnO with Tinuvin 5151 (T6) was also suitable, wherein their synergistic effect was demonstrated compared to the modifier itself (T2 and T4 treatments). The synergistic effect of UV stabilization treatments on wood has been confirmed in more works [42,72]; however, the results of experiments in this work show that this also depends on the underlying wood species. 

For the Douglas fir, the surface treatment effect on colour stabilization was not as noticeable as in larch (Figure 5 and Figure 6), although it is similar in colour (Figure 2, Table 1), likely due to specific extractives in larch wood [61]. More effective treatments were T2, T5 and T14, which contained ZnO nanoparticles either alone or in a mixture with other stabilizers. In addition, in motherore works [43,50,73,74], a significant effect of ZnO nanoparticles in decreasing the colour changes caused by UV radiation was observed.

Treatments T8, T9 and T10, using substances soluble only in organic solvents, were generally less effective. During UV and VIS radiation exposure, these substances cause a more pronounced yellowing of the underlying wood species, which was particularly noticeable in the lighter spruce (Figure 2 and Figure 7), and this change was statistically significant compared to untreated wood (Table 3). Tinuvin 1130, based on benzotriazoles (T9), appears to be a more appropriate treatment in this respect (Figure 4, Figure 5, Figure 6 and Figure 7, Table 3). The results show that it is important to also take into account the solvent base of the surface colour-stabilization treatments of wood during exposure to UV and VIS radiation.

### 3.2. Changes of Surface Roughness

**Figure 9:**
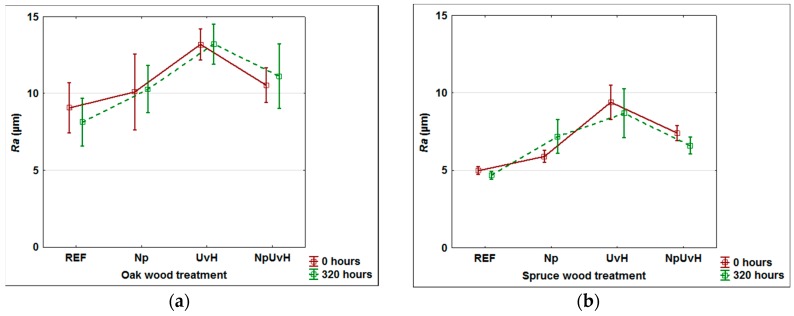
Surface roughness of oak (**a**) and spruce (**b**) wood before and after 320 h of artificial ageing. Reference (REF) without treatment; surfaces treated with water dispersion of nanoparticles (Np); surfaces treated with mixture of UV-stabilizers and HALS (UvH); surfaces treated with mixture of Np and UvH (NpUvH).

Higher changes in surface roughness of wood can positively affect the adhesion of applied topcoat layer, but the relationship is not fully clear [75,76]. An evaluation of the surface roughness of samples treated with UV and VIS radiation protective compounds is given in Figure 9. Oak wood as a hardwood species and spruce as a representative of tested softwoods were measured.

An increase in *Ra* (µm) was observed mainly if water solutions of UV-stabilizers and HALS were used as protective treatments. Using water-soluble treatments can cause an increase of wood fibres [16], which corresponds with these results. On the other hand, the application of dispersed nanoparticles, alone or in combination with UV and HALS stabilizers, is connected with the lower increase in the surface roughness. This can be explained by the penetration of nanoparticles into the open cells, which was observed under microscopic analyses (Figure 3). The effect of artificial ageing on surface roughness changes was negligible (Figure 9).

### 3.3. Final Discussion

This work is not aimed at monitoring chemical changes occurring in wood [14,31,39,66,77,78] or treated wood [26,27,32,35,42,79,80,81], as these have been thoroughly investigated and described in previous works. FTIR analyses show that degradation of lignin [31,77] is less significant and as rapid when protective layers containing UV-stabilizers, HALS or nanoparticles are applied on the wooden surfaces [26,27].

The aim was to describe the effect of several different treatments and their combinations on four underlying tree species, where a statistically significant effect was confirmed (Table 4). The wood species effect has been analysed in other works [33,44,71], but not on so many tree species or tested colour stabilization treatments. Several studies also describe the specific application of nanoparticle layers on wood [70,74,82] but, compared to the testing in this experiment, that seems more difficult to put into practice.

An important finding is that, based on the experimental results in this work, in some cases there was a significant synergistic effect of the combination of colour stabilizers. With regard to all of the tree species, this involved the T5 treatment whereby a mixture of UV-stabilizers based on benzotriazoles and HALS (Tinuvin 5151) in combination with nanoparticles achieved a much better effect, particularly in the oak, spruce and Douglas fir compared to non-combined substances (see Table 2 and Figure 4, Figure 5, Figure 6 and Figure 7). In larch, this effect was also achieved in a combination of T6 (Tinuvin 5151 in combination with ZnO nanoparticles). Compared to the work by Grüll et al. [61], this provides an opportunity to use, in addition to TiO_2_ nanoparticles (where good results were confirmed), ZnO nanoparticles in a suitable combination with UV stabilizers based on benzotriazoles and HALS. 

Compared to the above results (Figure 4, Figure 5, Figure 6 and Figure 7) and the test methodology used, the effect of colour changes tends to be emphasized by the higher moisture of the underlying wood species and higher temperature [83]. Adversely, prolonged test times do not lead to significantly higher colour changes in wood compared to the initial stages of photodegradation [5,54,55].

Decreasing the photodegradation of underlying wood species contributes not only to the reduction of lignin [39] and extractives [77] decomposition in surface layers; the degradation of cellulose, which contributes to micro-cracks in underlying wood species by reducing their mechanical properties [7], is also reduced. The most sensitive is amorphous fraction of cellulose [84].

The next step is to test these surface treatments in combination with surface treatment using top coating systems, whereby both their durability and colour consistency may be positively or negatively affected [1,21,26,28].

A promising result is the possibility of a better colour stabilization of larch (Figure 5) compared to other wood species (Figure 4, Figure 6 and Figure 7), because it is used on exterior façade elements, fences and other structures [61] thanks to its higher natural durability [58] and acceptable density. In combination with a suitable transparent coating system [2] that prevents the leaching of the UV-stabilizing surface treatment, the colour changes due to weathering would be greatly reduced. Preventing photodegradation of the underlying wood species would also extend its overall lifetime [1]. Another advantageous effect of the tested active treatment containing ZnO is its biocidal effect [85], which was also confirmed in the case of TiO_2_ [86].

## 4. Conclusions

The work is focused on reducing colour changes in four tree species, oak, larch, Douglas fir and spruce, via UV and VIS radiation using surface penetration treatments with UV stabilizers, HALS, nanoparticles ZnO and TiO_2_ and combinations thereof. Artificial accelerated ageing in Xenotest was used and the treatment efficiency was compared to untreated wood.

Based on the results, it is clear that the positive effect of a particular surface treatment depends on the type of underlying wood species. In several cases, the synergistic effect of the substances on the stabilization of wood by UV and VIS radiation was confirmed, when combinations of benzotriazoles, HALS and tested nanoparticles were applied. Decreasing the influence of radiation on wood surfaces was more effective when using reflected and scattered function of nanoparticles, absorption function of UV stabilizers, absorption and neutralization of free radicals by HALS as the combined effect, compared to the utilization of each protective treatment individually. Colour stabilization of oak is particularly difficult due to the frequent reaction and colouring of the surface layers after the application of UV stabilizers and nanoparticles. Spruce, a lighter wood species that is more susceptible to darkening and yellowing, was also positively affected by surface treatments in only a few cases compared to untreated surfaces. In contrast, the surface treatment of larch was more effective in several cases, and the synergistic effect of the combination of substances was also confirmed. The results were positive for the Douglas fir, but the colour stabilization was not as successful as in the case of larch. Generally, one of the most effective colour treatments for wood stabilization due to UV and VIS spectra was confirmed by testing the combination of benzotriazoles with HALS (Tinuvin 5151) and TiO_2_ and ZnO nanoparticles.

## Figures and Tables

**Table 1:** Density and initial colour parameters L*, a*, b* [65] of samples before testing.

Wood Species	Latin Name	Density (kg·m^−3^) at MC = 0%	L*	a*	b*
English oak	(*Quercus robur* L.)	712.3 (57.0)	66.5 (2.4)	6.8 (0.6)	19.8 (0.7)
Norway spruce	(*Picea abies* L. Karst)	485.6 (26.0)	82.5 (2.7)	4.5 (1.2)	21.3 (2.0)
European larch	(*Larix decidua* Mill.)	562.1 (38.9)	72.4 (3.2)	8.8 (2.3)	24.9 (2.5)
Douglas fir	(*Pseudotsuga menziesii* (Mirb.) Franco)	598.7 (8.7)	69.3 (1.3)	13.0 (1.5)	23.5 (0.6)

Note: MC means moisture content of wood; mean values are evaluated from 16 measurements; the numbers in parentheses are standard deviations. Density was measured on the 20 samples with dimensions 20 mm × 20 mm × 30 mm in oven dried state (103 ± 2) °C.

**Table 2:** Composition of surface penetration layers on wood.

Surface Treatment	Type of Substance (Ratio)	Composition (Manufacturer)	Solvent/Dispersion Base
**T1**	TiO_2_	4–8 nm nanoparticles (ROTI^®^nanoMETIC, Karlsruhe, Germany)	Distilled water
**T2**	ZnO	25 nm nanoparticles (ROTI^®^nanoMETIC, Karlsruhe, Germany)	Distilled water
**T3**	ZnO + TiO_2_ (1:1)	Combination of T1 and T2	Distilled water
**T4**	Tinuvin 5151	2-(2-hydroxyphenyl)-benzotriazoles with HALS (BASF, Ludwigshafen, Germany)	Distilled water
**T5**	Tinuvin 5151 + ZnO + TiO_2_ (1:1:1)	combination of T4 and T3	Distilled water
**T6**	Tinuvin 5151 + ZnO (1:1)	combination of T4 and T2	Distilled water
**T7**	Tinuvin 5151 + TiO_2_ (1:1)	combination of T4 and T1	Distilled water
**T8**	Tinuvin 123	bis(2,2,6,6-tetramethyl-1-octyloxypiperidin-4-yl)-1,10-decanedioate; 1,8-bis[(2,2,6,6-tetramethyl-4-((2,2,6,6-tetr amethyl-1-octyloxypiperidin-4-yl)-decan-1,10-dioyl)piperidin-1-yl)oxy]octane (BASF, Ludwigshafen, Germany)	Solution in butyl acetate
**T9**	Tinuvin 1130	on the base of b-[3-(2-H-Benzotriazole-2-yl)-4-hydroxy-5-*tert*.butylphenyl]-propionic acidpoly (ethylene glycol) 300-ester (BASF, Ludwigshafen, Germany)	Solution in butyl acetate
**T10**	Tinuvin 123 + Tinuvin 1130 (1:1)	Combination of T8 and T9	Solution in butyl acetate
**T11**	Sun Care 800	Mixture of synthetic resins, organic UV light stabilizers, IPBC fungicide (Bohme Switzerland)	Water solution
**T12**	Sun Care 900	UV light stabilizers in polymer dispersion (Bohme Switzerland)	Water solution
**T13**	Sun Care 800 + Tinuvin 5151 (3%)	Combination of T11 and added 3% of 2-(2-hydroxyphenyl)-benzotriazoles with HALS (BASF, Ludwigshafen, Germany)	Water solution and Tinuvin 5151 in distilled water
**T14**	Sun Care 800 + ZnO + TiO_2_ (3%; 1:1)	T11 and added 3% of 25 nm and 4–8 nm nanoparticles	Water solution and nanoparticles in distilled water
**REF**	Reference	Without	none

**Table 3:** Colour changes in tested samples after 320 h of accelerated ageing.

Type of Treatment		T1	T2	T3	T4	T5	T6	T7	T8	T9	T10	T11	T12	T13	T14	REF
**Oak**	∆L*	2.0 (2.4)	−5.1 (1.5)	−0.2 (4.8)	−1.4 (1.6)	1.1 (2.3)	0.0 (1.4)	2.1 (2.6)	−7.4 (1.7)	−5.3 (1.5)	−6.2 (2.6)	−1.0 (1.2)	−11.4 (1.4)	−2.2 (0.5)	−4.5 (1.7)	−5.7 (0.5)
	∆a*	−1.2 (1.5)	1.5 (0.5)	−0.4 (0.8)	0.4 (0.4)	−1.0 (0.8)	−1.1 (0.5)	−0.6 (1.3)	3.6 (0.7)	2.6 (0.3)	2.9 (1.4)	0.1 (0.5)	4.6 (0.4)	−2.2 (0.2)	0.8 (0.3)	3.1 (0.2)
	∆b*	−6.8 (1.8)	9.2 (1.4)	1.6 (1.1)	6.5 (1.1)	−3.4 (1.4)	4.4 (1.0)	−1.3 (2.0)	9.6 (0.5)	8.4 (0.4)	8.8 (1.6)	6.7 (0.4)	14.1 (0.7)	−3.7 (0.4)	8.0 (0.5)	6.8 (0.4)
	∆E*	7.5 (2.6)	10.8 (0.6)	4.7^•^ (1.5)	6.8^•^ (1.0)	4.5^•^S (0.7)	4.8^•^ (0.7)	3.8^•^ (1.8)	12.7^•^ (1.1)	10.3 (0.9)	11.3 (3.0)	6.9^•^S (0.6)	18.7^•^ (1.1)	4.8^•^ (0.4)	9.3 (0.9)	9.4 (0.3)
**Larch**	∆L*	−1.5 (3.5)	−8.8 (1.8)	−11.8 (2.7)	−8.1 (3.0)	−5.7 (2.5)	−5.0 (1.5)	−5.5 (3.5)	−12.1 (5.5)	−10.3 (1.6)	−9.8 (2.8)	−1.4 (1.9)	−5.0 (0.6)	4.4 (1.0)	−5.5 (1.9)	−12.0 (0.9)
	∆a*	−0.3 (0.9)	2.7 (1.1)	4.4 (1.2)	2.9 (1.5)	1.0 (1.4)	1.0 (0.6)	−1.2 (2.6)	5.0 (2.3)	0.6 (0.7)	4.0 (1.2)	1.3 (0.6)	−0.2 (0.7)	−2.2 (0.7)	0.3 (1.2)	2.5 (0.4)
	∆b*	−4.9 (2.5)	4.8 (2.3)	5.0 (1.1)	7.7 (2.3)	4.5 (3.7)	4.8 (2.0)	−3.9 (2.8)	12.0 (1.2)	2.9 (1.0)	9.9 (1.4)	4.8 (0.8)	7.4 (1.1)	−0.8 (1.1)	7.4 (0.8)	2.2 (0.5)
	∆E*	6.2^•^S (2.4)	10.6 (2.4)	13.6 (2.9)	11.8 (3.0)	8.3^•^ (2.2)	7.4^•^S (1.2)	7.7^•^S (3.7)	18.1^•^ (5.1)	10.8 (1.4)	14.7 (1.8)	5.4^•^ (1.6)	9.0 (1.1)	5.1^•^ (0.8)	9.4 (1.3)	12.5 (0.9)
**Douglas fir**	∆L*	−4.6 (0.3)	−6.4 (0.8)	−7.3 (1.2)	−7.5 (0.5)	−4.5 (2.4)	−6.9 (1.4)	−3.1 (1.3)	−9.6 (0.8)	−7.1 (0.6)	−9.3 (0.8)	−7.6 (0.3)	−8.1 (0.3)	−4.1 (0.3)	−2.8 (0.3)	−9.7 (0.1)
	∆a*	0.8 (0.4)	−2.3 (0.3)	−3.8 (0.5)	−1.7 (0.2)	−3.6 (1.0)	−3.7 (0.6)	−3.7 (0.3)	−0.9 (0.3)	1.6 (0.4)	−0.3 (0.3)	−2.4 (0.2)	−2.1 (0.2)	−5.8 (0.7)	−4.5 (0.1)	−0.7 (0.1)
	∆b*	−5.2 (0.5)	1.3 (1.2)	−2.7 (1.4)	7.0 (0.3)	−1.6 (1.3)	4.2 (0.7)	−5.6 (1.2)	6.5 (0.4)	5.8 (0.3)	7.8 (0.4)	5.0 (0.2)	6.8 (0.2)	−1.4 (1.0)	4.6 (0.3)	6.7 (0.1)
	∆E*	7.0^•^ (0.3)	7.0^•^S (0.9)	8.8^•^ (0.6)	10.4^•^ (0.4)	6.5^•^S (1.0)	8.9^•^ (1.0)	7.5^•^ (1.2)	11.6 (0.8)	9.3^•^ (0.6)	12.2 (0.4)	9.4^•^ (0.2)	10.8 (0.2)	7.3^•^ (0.2)	7.1^•^S (0.1)	11.8 (0.2)
**Spruce**	∆L*	−12.8 (1.2)	−12.6 (0.5)	−10.4 (3.1)	−9.1 (1.7)	−8.0 (1.9)	−9.8 (1.7)	−7.6 (3.6)	−14.7 (1.6)	−11.6 (1.2)	−11.4 (0.7)	−9.2 (0.7)	−12.5 (0.4)	−9.3 (0.6)	−7.3 (1.1)	−10.7 (0.6)
	∆a*	5.2 (0.7)	5.1 (0.3)	3.6 (1.4)	3.3 (0.9)	4.3 (0.9)	3.3 (1.0)	2.9 (2.2)	6.9 (0.6)	5.3 (0.4)	5.7 (0.4)	3.4 (0.3)	5.0 (0.1)	2.7 (0.5)	2.2 (0.5)	4.6 (0.2)
	∆b*	2.9 (2.1)	9.2 (0.9)	3.3 (3.4)	12.4 (2.6)	3.3 (1.6)	9.5 (1.6)	2.2 (3.7)	16.7 (1.2)	14.5 (0.4)	15.9 (0.9)	13.1 (0.8)	15.7 (0.4)	3.6 (1.5)	10.2 (0.7)	11.3 (0.7)
	∆E*	14.2 (1.8)	16.4 (0.9)	11.8^•^S (4.1)	15.7 (3.2)	9.8^•^S (2.0)	14.1 (2.4)	9.0^•^S (4.7)	23.3^•^ (1.9)	19.4 (1.1)	20.4^•^ (1.0)	16.4 (1.1)	20.7^•^ (0.5)	10.4^•^ (0.4)	12.8 (1.2)	16.2 (0.9)

Note: Symbol • means that the ∆E* values of surface treated samples were statistically significantly different compared to reference samples (REF) at a significance level of 95% after evaluation via the Tukey HSD test. Coloured are statistically significant effective treatments. Symbol “S” means the most suitable surface treatments without higher colour changes after their application and during artificial ageing (see also Figure 4, Figure 5, Figure 6 and Figure 7).

**Table 4:** Factorial ANOVA analyses of selected factors affecting the ΔE* values of treated wood surfaces after 320 h of accelerated ageing.

Categorical Factor	Sum of Squares	Degree of Freedom	Number of Squares	Fisher´s F Test	*p*-Value
Wood species (WS)	3632	3	1211	377.1	0.000 *
Type of Treatment (TT)	3919	14	280	87.2	0.000 *
WS*TT	1844	42	44	13.7	0.000 *

Note: WS*TT means influence of two factors together. Symbol * means: “statistically significant”.
